# Diabetic Neuropathy Evaluated by a Novel Device: Sural Nerve Conduction Is Associated with Glycemic Control and Ankle–Brachial Pressure Index in Japanese Patients with Diabetes

**DOI:** 10.3389/fendo.2017.00203

**Published:** 2017-08-15

**Authors:** Hidetaka Hamasaki, Yasuteru Hamasaki

**Affiliations:** ^1^Hamasaki Clinic, Kagoshima, Japan

**Keywords:** diabetic neuropathy, sural nerve, nerve conduction velocity, amplitude, glycemic control

## Abstract

**Background:**

Currently, no international diagnostic criteria for diabetic neuropathy (DN) have been established. Recently, a novel point-of-care sural nerve conduction device has been developed. We aimed to investigate associations between DN and clinical parameters related to the development and progression of DN by using this novel device.

**Methods:**

We conducted a retrospective observational study in patients with diabetes whose sural nerve functions were measured using DPN Check between January 2015 and October 2016. Multiple and logistic regression analyses were conducted to assess the associations of sural nerve conduction velocity (SNCV) and amplitude (SNAP) with clinical parameters related to DN.

**Results:**

A total of 740 patients were enrolled in this study. At baseline, 211 patients were diagnosed with DN by using DPN Check. The sensitivity, specificity, and positive likelihood ratio of DPN Check compared with ankle reflex as reference were 81%, 46%, and 1.5, respectively. Of these, 182 patients were followed up for approximately 1 year to measure changes in SNCV and SNAP. Both SNCV and SNAP were inversely associated with duration of diabetes, plasma glucose levels, and hemoglobin A1c levels at baseline, whereas these were positively associated with ankle–brachial index. Logistic regression analysis revealed that poor glycemic control was associated with increased risk of reduction in both SNCV [odds ratio = 1.570; 95% confidence interval (CI) = 1.298–1.898; *p* < 0.001] and SNAP (odds ratio = 1.408; 95% CI = 1.143–1.735; *p* = 0.001), and longer duration of diabetes was also significantly associated with an increased risk of reduction in both SNCV (odds ratio = 1.058; 95% CI = 1.032–1.084; *p* < 0.001) and SNAP (odds ratio = 1.049; 95% CI = 1.019–1.079; *p* = 0.001).

**Conclusion:**

Sural nerve functions were significantly associated with glycemic control and arteriosclerosis in patients with diabetes. DPN Check may be useful as a screening tool to identify DN in clinical practice.

## Introduction

Diabetic neuropathy (DN) develops with a background of prolonged hyperglycemia, which is a common diabetic complication, and impairs quality of life of patients with diabetes (1). Risk factors associated with the development and progression of DN includes poor glycemic control, duration of diabetes, hypertension, dyslipidemia, and smoking and drinking habits (2–4). Recent studies have debated regarding the influence of glycemic variability on DN as well as the average glucose and hemoglobin A1c (HbA1c) levels (5). Avoiding overt hyperglycemia and hypoglycemia may also be crucial in order to prevent the progression of DN. DN is known to be heterogeneous by its symptoms, onset, course, and types of neurological involvement (i.e., peripheral or autonomic); however, the most typical DN is a chronic, symmetrical, length-dependent sensorimotor polyneuropathy (1). Such diabetic sensorimotor polyneuropathy is frequently associated with retinal and renal microangiopathy, and usually develops on metabolic disturbances, but other causes should be excluded in the diagnosis (6). Currently, no international diagnostic criteria for DN have been established; however, the combination of symptoms, signs, and electrophysiological examination such as nerve conduction study is the most accurate in the diagnosis of DN (7). Simplified diagnostic criteria for diabetic polyneuropathy have been proposed for clinicians in Japan; patients must be diagnosed with diabetes (excluding those with neuropathies other than DN) and should also present with any two of the following three clinical manifestations: (a) sensory symptoms due to DN, (b) bilaterally absent or decreased ankle reflex, and (c) bilaterally decreased vibratory sensation in the medial malleolus (8). Although these diagnostic criteria are useful (sensitivity 68% and specificity 74% compared with nerve conduction study as reference) (8), clinicians might misdiagnose asymptomatic DN and might not have adequate time for neurological examination in routine practice. Recently, a novel point-of-care sural nerve conduction device has been developed; the device shows good reliability and validity (sensitivity 90.48% and specificity 79.17%), when evaluated using standard nerve conduction study as reference, and has the potential to be suitable for clinical applications (9, 10). In contrast to standard nerve conduction study, this method is less time consuming (approximately 3 min per examination) and user friendly. It may be useful as a screening test for DN in routine practice. On the other hand, to our knowledge, no studies have investigated the association between DN and clinical parameters in patients with diabetes using this handy device. Thus, the present study aimed to investigate and affirm the associations between DN and clinical parameters related to the development and progression of DN by using this novel device and discuss its applicability for diagnosing DN in routine clinical practice.

## Materials and Methods

### Study Design and Subjects

We conducted a retrospective observational study in patients with diabetes who were treated at Hamasaki Clinic. Between January 2015 and October 2016, a total of 740 individuals who underwent examination for DN were included. Patients younger than 20 years and those with neurological disorders other than DN were excluded. In addition, patients who could not undergo examination for DN because of lower extremity edema and injuries were excluded. Approximately 1 year after the initial examination for DN, patients were re-examined to determine whether their DN had deteriorated or improved. Since this study was an observational study, the opt-out method of obtaining informed consent was adopted. The patients were anonymized to protect their personal information. The study protocol was approved by the Medical Ethics Committee of the Japan Medical Association, Center for Clinical Trials (Reference No. 28-6), and the study was performed in accordance with the Declaration of Helsinki.

### Physical Examination and Medical History Taking

Patients were examined for ankle reflex on standing position on the knees. Two diabetologists separately examined ankle reflex, and they were blinded to results of ankle reflex each other. Patients were also asked about duration of diabetes and smoking and drinking habits at the first medical examination. Brinkman index (number of cigarettes per day multiplied by number of years) was calculated to quantify patients’ smoking habit (11).

### Anthropometric and Physiological Measurements

Height was measured using a rigid stadiometer (seca 217; seca Nihon Co., Ltd., Chiba, Japan). Weight was measured using calibrated scales (seca 899; seca Nihon Co., Ltd., Chiba, Japan). Body mass index (BMI) was calculated as body weight in kilograms divided by the square of body height in meters.

Blood pressure was measured in a seated position by using a sphygmomanometer (KM-382; KENZMEDICO Co., Ltd, Saitama, Japan). Arterial stiffness was examined by measuring ankle–brachial index (ABI) and toe–brachial index (TBI), and the brachial–ankle pulse wave velocity (baPWV) by using a pulse pressure analyzer (BP-203RPEIII; Omron Co., Ltd., Tokyo, Japan). Average values of right and left of ABI, TBI, and baPWV were analyzed. The coefficient of variation of R–R intervals (CVRR) on electrocardiogram (FCP-8600; FUKUDA DENSHI, Co., Ltd., Tokyo, Japan) was measured as a marker for cardiac autonomic neuropathy in patients with diabetes.

### Blood and Urinary Examination

We measured serum total cholesterol (Determiner L TC II, Kyowa Medex Co., Ltd., Tokyo, Japan), triglycerides (Determiner L TG II, Kyowa Medex Co., Ltd., Tokyo, Japan), high-density lipoprotein (HDL) cholesterol (Cholestest N HDL, SEKISUI MEDICAL Co., Ltd., Tokyo, Japan), low-density lipoprotein (LDL) cholesterol (Cholestest LDL, SEKISUI MEDICAL Co., Ltd., Tokyo, Japan), plasma glucose (PG), and HbA1c (HLC-723G9, TOSOH Co., Ltd., Tokyo, Japan). Serum C-peptide levels were measured using a chemiluminescent immunoassay kit (SIEMENS Healthcare Diagnostics, Co., Ltd., Tokyo, Japan). Urinary albumin creatinine ratio (UACR) (N-A TIA MicroALB, NITTOBO MEDICAL Co., Ltd., Tokyo, Japan) was also measured as a marker for diabetic nephropathy.

### Diabetic Neuropathy Assessment

**Figure 1:**
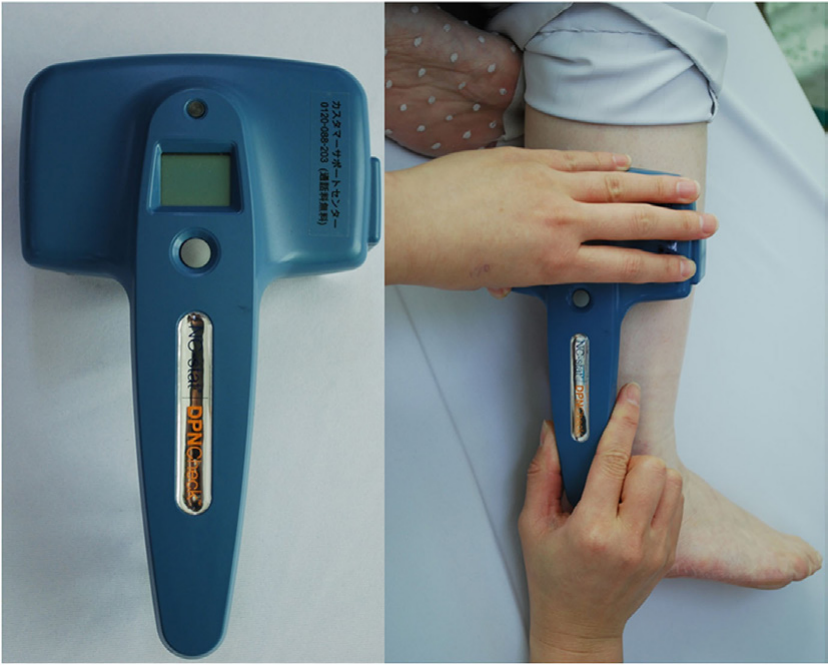
DPN Check measures sural nerve conduction velocity and amplitude with a disposable biosensor at a fixed distance from the stimulating probes, which are placed on the posterior side of the lateral malleolus.

The right lower limbs of patients were examined using a nerve conduction device named DPN Check (HDN-1000, Omron Co., Ltd., Tokyo, Japan) in the lateral decubitus position (Figure 1). The assessment was performed about 10 min after the physical examination. The examination room temperature was set at 25°C. This device measures sural nerve conduction velocity (SNCV) and amplitude (SNAP) with a disposable biosensor at a fixed distance of 9.22 cm from the stimulating probes. The stimulating probe is placed on the posterior side of the lateral malleolus of subjects, and examination is performed. The sural nerve was orthodromically stimulated 4–16 times within 10–20 s. The device also measured skin temperature because nerve conduction velocity is influenced by temperature. The device corrects SNCV for skin temperature between 23 and 28°C and prevents tests when skin temperatures are below 23°C. If SNAP is <2 μV or SNCV is undetectable, any results are automatically adjusted to 0 by the device. Subjects are diagnosed as normal or with neuropathy according to the cutoff values of both SNCV and SNAP adjusted by age and height in normative database: SNCV (cutoff value) = 99.4 − 0.16 × age − 0.23 × height; SNAP (cutoff value) = 11.2 − 0.099 × age (9). All examinations were performed by a skilled clinical technologist. The assessor was blinded to results of ankle reflex.

### Statistical Analysis

Statistical analyses were performed using SPSS version 24 (IBM Co., Ltd., Chicago, IL, USA). All values are expressed as mean ± SD. Multiple regression analyses adjusted for age, sex, and BMI were performed to test independent associations of both SNCV and SNAP with clinical parameters. Differences in continuous variables and categorical variables such as sex between patients with and without DN were analyzed by using *t* test and chi-square test, respectively. Moreover, logistic regression analyses were performed to analyze association between the presence of DN and clinical parameters and to determine odds ratio and 95% confidence intervals (CIs). We included age, sex, BMI, duration of diabetes, smoking habit, drinking habit, systolic blood pressure, diastolic blood pressure, total cholesterol, triglycerides, HDL cholesterol, LDL cholesterol, PG, and HbA1c in the logistic regression model. We conducted a *post hoc* sample size calculation using G*Power (http://www.gpower.hhu.de/). In the multiple regression model, observed *R*^2^ of HbA1c levels with SNCV and SNAP were 0.111 and 0.029, respectively. Cohen’s *f*^2^ effect size was calculated as follows: *R*^2^/(1 − *R*^2^). The number of predictors was 4, and the one-tailed alpha level is 0.025, the sample size calculation indicates that 364 subjects are needed for a power of 0.95. This suggests that our sample size had sufficient power to detect the observed association between sural nerve function and glycemic control. *p*-Values < 0.05, determined by performing a two-sided test, were considered statistically significant.

## Results

Of 740 patients (458 men and 282 women), 18 and 722 patients had type 1 and 2 diabetes, respectively. The mean patient age was 65.6 ± 12.0 years, the mean BMI was 24.1 ± 4.1 kg/m^2^, and the mean duration of diabetes was 15.3 ± 9.9 years. At baseline, 211 (28.5%) patients were diagnosed with DN by using DPN Check; Of these, 182 patients underwent DPN Check twice during the study period (average examination interval: 421 ± 51 days). Patient characteristics at baseline are listed in Table 1.

**Table 1:** Characteristics of patients at baseline.

Demographics	
*n*	740
Age (years)	65.6 (12)
Sex (male/female)	458/282
Types of diabetes (type 1/type 2)	18/722
Smoking habit (current or past/never/unknown)	239/472/29
Brinkman index	827 (548)
Drinking habit (yes/no/unknown)	348/363/29
Duration of diabetes (years)	15.3 (9.9)
**Anthropometric data**	
Height (cm)	161 (9.1)
Weight (kg)	62.7 (13.4)
BMI (kg/m^2^)	24.1 (4.1)
**Physiological and biochemical data**	
Systolic blood pressure (mmHg)	128.9 (17.2)
Diastolic blood pressure (mmHg)	70.7 (10.3)
Plasma glucose (mg/dl)	168.1 (70)
Plasma HbA1c (%)	7.8 (1.5)
Serum C-peptide (ng/ml)	1.3 (1.4)
Serum total cholesterol (mg/dl)	197.6 (35.2)
Serum triglyceride (mg/dl)	148.4 (103)
Serum high-density lipoprotein cholesterol (mg/dl)	52.1 (15.2)
Serum low-density lipoprotein cholesterol (mg/dl)	112.6 (29.6)
Urinary albumin creatinine ratio (mg/gCr)	37.5 (54.6)
Coefficient of variation of R–R intervals	2.64 (1.29)
Ankle–brachial index	1.15 (0.11)
Toe–brachial index	0.76 (0.17)
Brachial–ankle pulse wave velocity (cm/s)	1,778 (371)
Sural nerve conduction velocity	50.1 (10.5)
Sural nerve amplitude	10.2 (6.6)

*Data are expressed as means (SD) for continuous variables*.

*BMI, body mass index; HbA1c, hemoglobin A1c*.

In total, 680 patients underwent both ankle reflex and DPN Check. The concordance rate of DN diagnosis was 70.3%. However, among 382 patients without DN diagnosed by normal ankle reflex, 90 patients had decreased SNCV or SNAP. The sensitivity, specificity, and positive likelihood ratio of DPN Check compared with ankle reflex as reference were 81%, 46%, and 1.5, respectively (Table S1 in Supplementary Material).

SNCV was inversely associated with duration of diabetes, Brinkman index, PG levels, HbA1c levels, and serum triglyceride levels at baseline and changes in SNCV, whereas it was positively associated with CVRR and ABI. On the other hand, SNAP was inversely associated with duration of diabetes, PG levels, and HbA1c levels at baseline and changes in SNAP, whereas it was positively associated with ABI (Table 2).

**Table 2:** Associations between sural nerve functions and clinical parameters.

	SNCV		SNAP	
	β	*p*-Value	β	*p*-Value
Duration of diabetes	−0.216	<0.001	−0.19	<0.001
Brinkman index	−0.19	0.018	−0.028	0.73
Systolic blood pressure	−0.065	0.086	−0.046	0.21
Diastolic blood pressure	0.018	0.66	0.047	0.23
Plasma glucose	−0.078	0.036	−0.097	0.007
Plasma HbA1c	−0.155	<0.001	−0.148	<0.001
Serum total cholesterol	0.052	0.23	0.039	0.36
Serum triglyceride	−0.085	0.029	0.025	0.51
Serum HDL cholesterol	0.038	0.33	0.025	0.51
Serum LDL cholesterol	0.065	0.083	0.053	0.14
Serum C-peptide	0.178	0.18	0.242	0.057
Urinary albumin creatinine ratio	−0.063	0.17	−0.065	0.14
CVRR	0.336	0.016	0.159	0.25
ABI	0.191	0.001	0.147	0.007
TBI	0.104	0.071	0.061	0.27
baPWV	−0.048	0.45	0.035	0.56
⊿SNCV	−0.31	<0.001	0.079	0.3
⊿SNAP	−0.084	0.26	−0.519	<0.001

*The multiple regression model was adjusted for age, sex, and body mass index (BMI). Each variable displayed in a separate row of the table was adjusted for age, sex and BMI*.

*SNCV, sural nerve conduction velocity; SNAP, sural nerve amplitude; HbA1c, hemoglobin A1c; HDL, high-density lipoprotein; LDL, low-density lipoprotein; CVRR, coefficient of variation of R–R intervals; ABI, ankle–brachial index; TBI, toe–brachial index; baPWV, brachial–ankle pulse wave velocity. ⊿ means annual changes in SNCV and SNAP*.

In patients with DN, duration of diabetes was longer, PG, HbA1c levels, and UACR were higher, and ABI and TBI were lower than those in patients without DN (Table 3).

**Table 3:** Comparison of clinical data between patients with and without diabetic neuropathy.

	With diabetic neuropathy	Without diabetic neuropathy	*p*-Value
Age (years)	64.2 (13.1)	66.1 (11.4)	0.057
Duration of diabetes (years)	17.4 (10.1)	14.5 (9.7)	<0.001
Sex (male/female)	121/90	337/192	0.11
Height (cm)	161 (9.7)	161.1 (8.8)	0.93
Weight (kg)	63.3 (14.1)	62.5 (13.1)	0.42
BMI (kg/m^2^)	24.4 (4.8)	23.9 (3.7)	0.22
Brinkman index	841 (572)	821 (539)	0.82
Systolic blood pressure (mmHg)	130 (18)	128.5 (16.9)	0.28
Diastolic blood pressure (mmHg)	70.2 (11.3)	70.8 (9.9)	0.53
Plasma glucose (mg/dl)	184.6 (82.8)	161.5 (63.1)	<0.001
Plasma HbA1c (%)	8.4 (1.7)	7.6 (1.3)	<0.001
Total cholesterol (mg/dl)	194 (38)	199 (34)	0.14
Triglycerides (mg/dl)	152.6 (105.3)	146.7 (102.1)	0.49
HDL cholesterol (mg/dl)	51.6 (16.6)	52.3 (14.6)	0.57
LDL cholesterol (mg/dl)	110.1 (30.9)	113.7 (29)	0.14
Urinary albumin creatinine ratio (mg/gCr)	53.7 (64.9)	33 (50.6)	0.003
CVRR	2.28 (1.12)	2.83 (1.35)	0.12
ABI	1.12 (0.15)	1.16 (0.09)	0.005
TBI	0.71 (0.17)	0.78 (0.16)	<0.001
baPWV (cm/s)	1,828 (423)	1,758 (347)	0.13
SNCV (m/s)	40.9 (14.8)	53.9 (4.3)	<0.001
SNAP (μV)	5.9 (5.1)	11.9 (6.4)	<0.001

*Values are expressed as means (SD) except for sex*.

*BMI, body mass index; HbA1c, hemoglobin A1c; HDL, high-density lipoprotein; LDL, low-density lipoprotein; CVRR, coefficient of variation of R–R intervals; ABI, ankle–brachial index; TBI, toe–brachial index; baPWV, brachial–ankle pulse wave velocity; SNCV, sural nerve conduction velocity; SNAP, sural nerve amplitude*.

Furthermore, logistic regression analysis revealed that higher HbA1c levels at baseline were significantly associated with an increased risk of reduction in both SNCV [odds ratio = 1.570; 95% confidence interval (CI) = 1.298–1.898; *p* < 0.001] and SNAP (odds ratio = 1.408; 95% CI = 1.143–1.735; *p* = 0.001), and longer duration of diabetes was also significantly associated with an increased risk of reduction in both SNCV (odds ratio = 1.058; 95% CI = 1.032–1.084; *p* < 0.001) and SNAP (odds ratio = 1.049; 95% CI = 1.019–1.079; *p* = 0.001). In addition, age was associated with a decreased risk of reduction in SNCV, and BMI was associated with an increased risk of reduction in SNAP. However, no significant associations were found between smoking and drinking habits, blood pressure, and serum lipid profile and SNAP or SNCV (Table 4).

**Table 4:** Logistic regression analyses for age, sex, BMI, duration of diabetes, smoking, drinking, blood pressure, lipid profile, and glycemic control.

	SNCV			SNAP		
	Adjusted odds ratio	95% CI	*p*-Value	Adjusted odds ratio	95% CI	*p*-Value
Age	0.972	0.950–0.994	0.012	0.975	0.949–1.001	0.063
Sex						
Men	0.655	0.383–1.121	0.12	0.734	0.386–1.395	0.35
Women	Reference			Reference		
Body mass index	0.979	0.924–1.036	0.46	1.072	1.004–1.143	0.036
Duration of diabetes	1.058	1.032–1.084	<0.001	1.049	1.019–1.079	0.001
Smoking habit						
Current or past	1.222	0.738–2.024	0.44	1.010	0.551–1.853	0.97
Never	Reference			Reference		
Drinking habit						
Yes	0.980	0.603–1.594	0.94	0.671	0.372–1.208	0.18
No	Reference			Reference		
Systolic blood pressure	1.011	0.994–1.027	0.2	1.019	0.999–1.038	0.057
Diastolic blood pressure	0.992	0.963–1.021	0.57	0.985	0.952–1.020	0.41
Serum total cholesterol	1.029	0.784–1.350	0.84	1.031	0.692–1.535	0.88
Serum triglyceride	0.993	0.941–1.049	0.81	0.995	0.919–1.077	0.9
Serum HDL cholesterol	0.968	0.738–1.271	0.82	0.971	0.651–1.447	0.89
Serum LDL cholesterol	0.962	0.733–1.262	0.78	0.968	0.649–1.442	0.87
Plasma glucose	0.997	0.993–1.001	0.14	0.998	0.993–1.003	0.37
Plasma HbA1c	1.570	1.298–1.898	<0.001	1.408	1.143–1.735	0.001

*CI, confidence intervals; SNCV, sural nerve conduction velocity; SNAP, sural nerve amplitude; HDL, high-density lipoprotein; LDL, low-density lipoprotein; HbA1c, hemoglobin A1c*.

## Discussion

We demonstrated that both SNCV and SNAP, measured using a novel device for sural nerve function, were significantly associated with duration of diabetes, glycemic control and arteriosclerosis in patients with diabetes. These findings are consistent with previous reports (12, 13); however, the lack of uniformity of DN measurement such as questionnaire, ankle reflex, monofilament testing, and vibration perception threshold makes it difficult to conclude whether various therapies are adequately effective in improving DN (14). In this regard, DPN Check exhibits high sensitivity and specificity for standard nerve conduction studies (9, 10) and can be used without special training in standard nerve conduction studies; thus, it should be reliable for evaluating DN in clinical practice. To our knowledge, this is the first study to show that DN evaluated by DPN Check is significantly associated with clinical parameters in Japanese patients with diabetes.

The evidence that intensive glycemic control is effective for preventing progression of DN in patients with type 1 diabetes is clear (14). The Diabetes Control and Complications Trail/Epidemiology of Diabetes Interventions and Complications trials, wherein DN was assessed by a standardized evaluation method, revealed that the prevalence of DN substantially increased in the conventional therapy group (from 5 to 17%) after 6.5 years of follow-up compared with the intensive therapy group (from 7 to 9%) (15–17). On the other hand, the results of randomized controlled trials in patients with type 2 diabetes are controversial. The Kumamoto study (18) showed that intensive glycemic control (achieved HbA1c of 7.4%) prevented the progression of DN, and the Action to Control Cardiovascular Risk in Diabetes trial (19) reported that intensive glycemic intervention (target HbA1c of <6%) reduced the incidence of DN compared with standard glycemic control (target HbA1c of 7.0–7.9%); however, the United Kingdom Prospective Diabetes Study (UKPDS) (20), Veterans Affairs Diabetes Trial (21), and Steno-2 trial (22) did not report any beneficial effects on the development or progression of DN. These large-scale clinical trials did not include DN as a primary outcome; thus, the design and subjects of these studies may not be suitable to investigate effects of glycemic control on DN. However, the heterogeneity of the method for evaluating DN may be the primary barrier to clarify the effect of glycemic control on DN progression (14). In patients with DN, nerve conduction studies revealed abnormalities, such as reduced amplitude, slowed conductive velocity, or prolonged latent phase (23). Distal, symmetrical, length-dependent sensorimotor polyneuropathy, which is the most common pattern of DN, is associated with progressive distal axonopathy (24). The pathogenic mechanism of DN is multifactorial; however, enhanced polyol pathway, increased advanced glycation end products, increased oxidative stress, and cytokine release induced by hyperglycemia are the causative factors for DN (25). Considering the pathophysiological mechanism of DN, it is useful to measure SNAP and SNCV as well as glycemic control in clinical practice.

Obesity and metabolic syndrome, particularly elevated triglycerides, are significantly associated with polyneuropathy irrespective of diabetes (26, 27). Dyslipidemia and increased nerve oxidative stress led to development of neuropathy in non-diabetic mice (28). Smith and Singleton (29) also reported that hypertriglyceridemia increased the risk of neuropathy and triglycerides were related to impairment of small unmyelinated fibers in patients with type 2 diabetes. Regular smoking also increases the incidence of DN independent of glycemic control (2) *via* microangiopathy due to oxidative stress (30). The findings of this study may represent the harmful effects of smoking on sensory nerve conduction velocity, concomitant with hyperglycemia and dyslipidemia.

Aging is also an independent risk factor for the development of DN (31); however, aging was associated with a decreased risk of reduction in SNAP. Although the underlying cause is unknown, older patients with diabetes might have already suffered from progressed DN which does not change in only 1 year. On the other hand, the positive association between SNCV and CVRR, which is an index of parasympathetic nervous activity, is convincing. Cardiac autonomic nervous dysfunction is a type of DN (1), and CVRR is lower in diabetic patients with polyneuropathy than in those without (32). However, the reason why we did not observe significant associations of SNAP with serum lipid profile, smoking status, and CVRR, and the reason why BMI was not associated with an increased risk of reduction in SNCV remain unknown and warrants further investigation.

Other notable findings of this study include positive associations of both SNCV and SNAP with ABI, an inverse association of baseline SNCV with changes in SNCV, and inverse association of baseline SNAP with changes in SNAP. McDermott et al. reported that peripheral artery disease measured by ABI impaired peripheral nerve function (33). In a prospective cohort study, Cardoso et al. demonstrated that increased aortic stiffness at baseline predicts the development or progression of DN (34). These findings suggest that diabetic micro- and macro-angiopathy is a causative factor for DN. Moreover, prevention of progression of arteriosclerosis is essential for managing DN. The inverse association between sural nerve function (both SNCV and SNAP) at baseline and changes in sural nerve function 1 year later suggests that DN rapidly deteriorates once DN develops. A systematic review revealed that several treatments such as α-lipoic acid, opioids, botulinum toxin A, and mexidol in addition to antidepressants and antipsychotics are effective in improving DN symptoms (35); however, there is no current evidence regarding the effective therapy for enhancing peripheral nerve function in patients with DN. The prevention of development of DN is crucial, and our findings indicate that early detection of DN and appropriate intervention during early stages, preferably prediabetes (36), will be needed.

This study had several limitations. First, there are some missing values because of the study design. Second, we did not measure SNCV and SNAP bilaterally. However, DN begins focally, but most patients eventually develop bilateral lower extremity symptoms (24). The mean duration of diabetes of patients with DN was 17.4 ± 10.1 years in the present study; thus, most patients were expected to have similar bilateral sural nerve impairment. Third, we did not evaluate patients’ symptoms and certain diagnostic indicators other than ankle reflex such as vibration perception threshold. We could not evaluate the diagnostic accuracy of DPN Check because clinical findings; symptoms, signs, and standard nerve conduction study were insufficient (7). However, as shown in previous studies (9, 10), DPN Check had a high sensitivity and a relatively low specificity in this study. This device can rule out the presence of DN if the test is negative; however, it is not suitable for the definitive diagnosis of DN. Fourth, there should have been a heterogeneity of physical examination between diabetologists in this study. Fifth, *post hoc* sample size calculation is also a limitation. Finally, according to the National Health and Nutrition Survey in 2008, the prevalence of DN in Japanese patients with diabetes was 11.8% (37); thus, we cannot generalize the results to other primary care populations. The discrepancy in the prevalence of DN may attribute to our clinical setting which is a clinic specializing in the management of diabetes. Despite these limitations, we could demonstrate that sural nerve functions evaluated by the novel device DPN Check were significantly associated with glycemic control and arteriosclerosis in patients with diabetes. To ensure these associations and assess the effects of diabetes treatment on DN, additional studies, preferably randomized controlled trials that include peripheral nerve function as a primary outcome, are required.

In conclusion, findings of this study suggest that early initiation of treatment for diabetes is essential for preventing the progression of DN. The factors associated with DN were duration of diabetes, glycemic control, and ABI. This study also showed the utility of DPN Check in clinical practice, which may be useful as a screening tool to identify DN.

## Ethics Statement

Since this study was an observational study, the opt-out method of obtaining informed consent was adopted. The patients were anonymized to protect their personal information. The study protocol was approved by the Medical Ethics Committee of the Japan Medical Association, Center for Clinical Trials (Reference No. 28-6), and the study was performed in accordance with the Declaration of Helsinki.

## Author Contributions

HH performed the study, conducted the data analyses, drafted the manuscript, revised the manuscript, and critically reviewed the manuscript and the scientific interpretations of study results. All authors read and approved the final manuscript.

## Conflict of Interest Statement

The authors declare that the research was conducted in the absence of any commercial or financial relationships that could be construed as a potential conflict of interest.
