# Supplementary material for: Diabetic Neuropathy Evaluated by a Novel Device: Sural Nerve Conduction Is Associated with Glycemic Control and Ankle–Brachial Pressure Index in Japanese Patients with Diabetes
Source: Front Endocrinol (Lausanne). 2017 Aug 15;8:203. doi: 10.3389/fendo.2017.00203 (PMC5559425; doi:10.3389/fendo.2017.00203)
Supplement: Supplementary file 1 [file Table_1.DOCX]

**Supplementary Table 1.** 2x2 contingency table for the diagnostic accuracy of DPN Check compared with ankle reflex.

|  | | **Ankle reflex** | | Total |
| --- | --- | --- | --- | --- |
|  |  | **Normal** | **Decreased** |  |
| **DPN Check** | **Normal** | 382 | 112 | 494 |
|  | **Decreased** | 90 | 96 | 186 |
| Total | | 472 | 208 | 680 |
